# Zinc deficiency and advanced liver fibrosis among HIV and hepatitis C co-infected anti-retroviral naïve persons with alcohol use in Russia

**DOI:** 10.1371/journal.pone.0218852

**Published:** 2019-06-27

**Authors:** Joshua A. Barocas, Kaku So-Armah, Debbie M. Cheng, Dmitry Lioznov, Marianna Baum, Kerrin Gallagher, Daniel Fuster, Natalia Gnatienko, Evgeny Krupitsky, Matthew S. Freiberg, Jeffrey H. Samet

**Affiliations:** 1 Section of Infectious Diseases, Boston Medical Center, Boston, Massachusetts, United States of America; 2 Boston University School of Medicine, Boston, Massachusetts, United States of America; 3 Boston University School of Public Health, Boston, Massachusetts, United States of America; 4 First Pavlov State Medical University, St. Petersburg, Russia; 5 Smoridintsev Research Institute of Influenza, St. Petersburg, Russia; 6 Florida International University, Robert Stempel College of Public Health and Social Work, Miami, Florida, United States of America; 7 Hospital Universitari Germans Trias I Pujol, Badalona, Spain; 8 Section of General Internal Medicine, Boston Medical Center, Boston, Massachusetts, United States of America; 9 V.M. Bekhterev National Medical Research Center for Psychiatry and Neurology, St. Petersburg, Russia; 10 Vanderbilt University Medical Center, Nashville, Tennessee, United States of America; Medizinische Fakultat der RWTH Aachen, GERMANY

## Abstract

**Background and aims:**

Liver disease in people living with HIV co-infected with hepatitis C virus is a source of morbidity and mortality in Russia. HIV accelerates liver fibrosis in the setting of HCV co-infection and alcohol use. Zinc deficiency is common among people living with HIV and may be a factor that facilitates the underlying mechanisms of liver fibrosis. We investigated the association between zinc deficiency and advanced liver fibrosis in a cohort of HIV/HCV co-infected persons reporting heavy drinking in Russia.

**Methods:**

This is a secondary data analysis of baseline data from 204 anti-retroviral treatment naïve HIV/HCV co-infected Russians with heavy drinking that were recruited into a clinical trial of zinc supplementation. The primary outcome of interest in this cross-sectional study was advanced liver fibrosis. Zinc deficiency, the main independent variable, was defined as plasma zinc <0.75 mg/L. Exploratory analyses were performed examining continuous zinc levels and fibrosis scores. Analyses were conducted using multivariable regression models adjusted for potential confounders.

**Results:**

The prevalence of advanced liver fibrosis was similar for those with zinc deficiency compared to those with normal zinc levels, (27.7% vs. 23.0%, respectively). We did not detect an association between zinc deficiency and advanced liver fibrosis in the adjusted regression model (aOR: 1.28, 95% CI: 0.62–2.61, p = 0.51) nor in exploratory analyses.

**Conclusions:**

In this cohort of Russians with HIV/HCV co-infection, who are anti-retroviral treatment naïve and have heavy alcohol use, we did not detect an association between zinc deficiency or zinc levels and advanced liver fibrosis.

## Background

Russia is currently experiencing converging epidemics of HIV, hepatitis C virus (HCV), and alcohol use disorder [1]. Liver disease in people living with HIV (PLWH) is a common source of morbidity and mortality in Russia [2]. Among Russians who are living with HIV and also use alcohol, liver-related causes are the second most common cause of death, following HIV-related diseases [3]. While HIV directly contributes to liver fibrosis, this effect is mitigated by the use of anti-retroviral treatment (ART) and much of the advanced fibrosis progression and liver-related death in this population is attributable to both alcohol as well as hepatotropic viruses such as HCV [4–6]. Recent estimates of HCV prevalence in Russia suggest that 3–4% of the general population is infected [7, 8]. Injection drug use accounts for most HIV and HCV co-infection in Russia. Approximately 20–25% of people who inject drugs (PWID) have HIV [9, 10] and more than two-thirds of PWID in Russia are infected with HCV [9, 11]. As a result, HIV/HCV co-infected PWID in Russia, where alcohol use is also highly prevalent [12], are at substantial risk of cirrhosis, hepatocellular carcinoma, and liver-related mortality.

Additionally, an estimated 8% of the Russian population is zinc deficient [13]. Zinc is an easily supplemented, essential nutrient and an antioxidant that is needed for, among other things, proper immune function [14]. Specifically, zinc deficiency reduces the generation of T cells and depresses both humoral and cell-mediated immunity, thus, accelerating HIV disease [15]. Notably, zinc deficiency has been found in greater than 50% of PLWH [16–18]. Zinc deficiency may be an independent risk factor for fibrosis progression in HCV liver disease. Experimental studies suggest that this association may be a consequence of zinc’s effects on the activity and availability of proteins, including the enzymes needed for the production and destruction of collagen [19–21]. Since collagen is a necessary component in liver fibrosis, zinc deficiency may directly contribute to advancing fibrosis. Moreover, fibrosis progression is influenced by oxidative stress and lower antioxidants. As an antioxidant, zinc has the capacity to reduce oxidative stress and, thus, slow the rate of fibrosis progression. In fact, zinc supplementation was found to reduce the incidence of hepatocellular carcinoma in persons with chronic HCV infection [22].

The impact of zinc on liver fibrosis among HIV/HCV co-infected persons is not well understood. One initial study demonstrated that low zinc levels are associated with increases in FIB-4 scores in co-infected persons [23]. To date, no studies have assessed the association of zinc deficiency and liver fibrosis outside of the U.S. among HIV/HCV co-infected persons. Given the high prevalence of HIV, HCV, alcohol use, and zinc deficiency in Russia, this setting presents an opportunity to explore research in this domain. We, therefore, aimed to investigate whether zinc deficiency is associated with advanced liver fibrosis in a cohort of HIV/HCV co-infected persons with heavy drinking in Russia.

## Patients and methods

### Study design

We conducted a cross-sectional analysis of data from the *Zinc for INflammation and Chronic disease in HIV (ZINC HIV)* clinical trial to explore the association between zinc deficiency and liver fibrosis.

### Study participants

Participants were recruited for the *ZINC HIV* trial between October 2013 and June 2015 from clinical HIV and addiction care sites, non-clinical sites and snowball recruitment to participate in a double-blinded randomized controlled trial in St. Petersburg, Russia to assess the efficacy of zinc supplementation vs. placebo on HIV morbidity [24]. This study recruited 254 individuals who met the following eligibility criteria: 1) age 18 to 70 years old; 2) HIV-seropositive; 3) provided information for at least two contacts; 4) had a stable address within St. Petersburg or districts within 100 kilometers of the city; 5) possessed a home or a mobile phone; 6) had recent history of heavy alcohol consumption (i.e., National Institute on Alcohol Abuse and Alcoholism [NIAAA] risky drinking criteria: > 4 standard drinks in a day [or > 14 standard drinks/week] for men and > 3/day [or 7/week] for women); and 7) were anti-retroviral treatment (ART) naïve at the time of enrollment. Participants were excluded if they were not fluent in Russian; had a cognitive impairment resulting in inability to provide informed consent; or were breastfeeding or pregnant. Not all individuals in the randomized controlled trial were HCV-infected. For the current cross-sectional analysis, we included eligible participants who also had a positive qualitative HCV RNA test at baseline.

The study was approved by the institutional review boards of Boston University School of Medicine/Boston Medical Center and First St. Petersburg Pavlov State Medical University. All participants provided written informed consent and were reimbursed the equivalent of USD $33 for completion of the baseline visit.

### Assessments

Data were collected through in-person interviews and blood collection. For this analysis, baseline data were used. Most laboratory assays were performed at St. Petersburg Pasteur Institute Central Clinical Diagnostic Laboratory except for zinc level testing, which was conducted at the ImmunoBioService laboratory in St. Petersburg. For zinc level testing, blood was collected in trace mineral free heparin tubes.

### Outcomes

Advanced liver fibrosis at baseline was the primary outcome of interest. This was defined using validated thresholds of Fibrosis-4 (FIB-4), aspartate aminotransferase (AST) to Platelet Ratio Index (APRI) and elastography (Fibroscan). FIB-4 is calculated using age, AST, aspartate aminotransferase (ALT), and platelet count. A FIB-4 score >3.25 has positive predictive value of >80% with a specificity of >98% for predicting advanced fibrosis [25]. APRI is the ratio of AST to platelets. APRI≥1.5 has an 88% positive predictive value and 95% specificity for predicting advanced fibrosis [26]. Elastography is an imaging technique to measure liver stiffness. Elastography values ≥10.5 kPa have 95% sensitivity and 63% specificity for predicting increased risk of clinical liver-related outcomes (i.e., decompensation) [27]. Only participants with FIB-4 values in the indeterminate range (1.45–3.25) were further evaluated by elastography. The final definition of the primary outcomes was FIB-4>3.25 or APRI≥1.5 or elastography≥10.5kPa. A secondary analysis was conducted modeling FIB-4 as a continuous variable.

### Main independent variable

The primary independent variable was zinc deficiency at baseline from the larger randomized trial, therefore, data do not reflect the use of zinc supplementation or placebo in the larger trial. We defined zinc deficiency as plasma zinc concentration <0.75 mg/L. This is consistent with previous studies that have assessed the impact of zinc deficiency on FIB-4 (23). In secondary analyses, we categorized zinc concentration into tertiles. We performed additional exploratory analyses in which zinc concentration was treated as a continuous variable.

### Covariates

Potential confounders were selected based on the literature and clinical knowledge. Confounders included sociodemographic characteristics such as age, gender, and body mass index (BMI); HIV disease characteristics such as HIV viral load, CD4 count, and self-reported time since diagnosis; self-reported hepatitis B virus (HBV) status; past 30-day injection drug use; and alcohol consumption [28]. Self-reported alcohol use was assessed to determine Diagnostic and Statistical Manual of Mental Disorders, 4th Edition diagnoses of abuse or dependence in the past year that was analyzed as a dichotomous variable.

### Statistical analysis

We used descriptive statistics to characterize the demographic and clinical characteristics of the analytic sample, overall and stratified by zinc deficiency. We compared groups using chi square and Fisher’s exact tests for categorical variables and t-tests and Wilcoxon tests for continuous variables, as appropriate. We estimated the proportion with advanced fibrosis by zinc deficiency status (<0.75 mg/L vs ≥0.75 mg/L). Correlations between independent variables and covariates were assessed using Spearman correlation and no pair of variables included in the regression models had a correlation > 0.40. We constructed a series of multivariable logistic regression models to estimate the association between zinc level and the outcome of interest, advanced liver fibrosis, controlling for potential confounding factors. We first fit a partially adjusted model that included time since HIV diagnosis, alcohol abuse or dependence, log_10_ HIV viral load, and CD4 count. We then fit a fully adjusted model that additionally included age, gender, hepatitis B co-infection, and body mass index. This fully adjusted model that included all potential confounders was considered the final model.

Secondary analyses were conducted with zinc level categorized into tertiles (lowest tertile: zinc concentration ≤0.7646 mg/L; middle tertile: zinc concentration >0.7646 mg/L and ≤0.9754 mg/L; highest tertile: zinc concentration >0.9754 mg/L) and the effect on advanced liver fibrosis was examined using the same approach described above. Lastly, we used generalized additive models (GAMs) [29] to explore modeling zinc concentrations and liver fibrosis based on FIB-4 score as continuous variables. Based on the GAMs, we subsequently used multiple linear regression models to evaluate the relationship between zinc concentrations and FIB-4 scores. Two-tailed tests and an alpha level of 0.05 were used for all tests. All analyses were performed using SAS version 9.3 (SAS Institute, Inc, NC, USA).

## Results

### Participant characteristics

Among the 254 trial participants, 204 met inclusion criteria for the current study. Among the 204 participants included in the final analytic sample, 65 (32%) had a plasma zinc level <0.75mg/L (Table 1). Among those with zinc deficiency, median zinc level was 0.6mg/L (interquartile range (IQR) 0.5–0.7) and among those with normal zinc levels, median was 1.0mg/L (IQR 0.9–1.2). Overall, participants had the following characteristics: median age of 33 years; 25% female; diagnosed with HIV for a mean of 7.5 years; mean log_10_ HIV viral load of 4.3; and mean BMI of 22.9 kg/m^2^. Injection drug use was reported in the past 30 days by 42% and 88% met criteria for alcohol abuse or dependence in the past year. Zinc supplementation or multivitamin use at baseline was reported by 9% (19/204) of participants, almost all (17/19) endorsed multivitamin use without separate zinc supplements. Among those with and without zinc deficiency, the following were common and similar: past 30-day injection drug (41% vs. 42%, p = 1.0); heavy alcohol use (95% vs. 92%, p = 0.56); alcohol abuse or dependence (88% vs 89%, p = 0.82); and self-reported HBV positivity (32% vs 36%, p = 0.54).

**Table 1:** Patient demographic and clinical characteristics of HIV and HCV co-infected ART naïve Russians.

	Overall (n = 204)	Zinc deficiency^†^ (n = 65)	Normal zinc levels (n = 139)	*p* value
Age, mean (SD)	33.6 (5.2)	33.9 (5.5)	33.5 (5.0)	0.58
Gender, male, (%)	154 (75.5%)	48 (73.8%)	106 (76.3%)	0.71
Time since HIV diagnosis, years, mean (SD)	7.5 (4.8)	8.1 (4.6)	7.2 (4.9)	0.23
CD4 count, median (25^th^, 75^th^ percentile)	463.7 (294.2, 700.1)	423.4 (256.8, 709.7)	471.2 (304.0, 697.5)	0.60
CD4 count (%)				
<350	68 (33.3%)	25 (38.5%)	43 (30.9%)	0.31
350–500	48 (23.5%)	17 (26.2%)	31 (22.3%)	
>500	88 (43.1%)	23 (35.4%)	65 (46.8%)	
HIV viral load (Log_10_ IU/mL), median (25^th^, 75^th^ percentile)	4.4 (3.6, 5.1)	4.4 (3.7, 4.8)	4.4 (3.4, 5.2)	0.52
Hepatitis B antibody positive, yes (%)	72 (35.3%)	21 (32.3%)	51 (36.7%)	0.54
Body Mass Index, mean (SD)	22.9 (3.1)	22.8 (3.1)	22.9 (3.1)	0.92
Alcohol abuse/dependence, yes (%)	179 (88.2%)	58 (89.2%)	121 (87.7%)	0.82
Alcohol consumption past 30 days^‡^				
Heavy drinking	190 (93.1%)	62 (95.4%)	128 (92.1%)	0.56
Moderate drinking	14 (6.9%)	3 (4.6%)	11 (7.9%)	
Current cocaine use, yes (%)	4 (2.0%)	1 (1.6%)	3 (2.2%)	1
Past 30-day injection drug use, yes (%)	84 (41.8%)	26 (41.3%)	58 (42.0%)	1
Advanced fibrosis^§^, yes (%)	50 (24.5%)	18 (27.7%)	32 (23.0%)	0.47
Zinc level (mg/L), median (25^th^, 75^th^ percentile)	0.9 (0.7, 1.0)	0.6 (0.5, 0.7)	1.0 (0.9, 1.2)	< .0001^§§^

SD, standard deviation

^†^Zinc deficiency defined as plasma Zinc <0.75 mg/L group

^‡^National Institute on Alcohol Abuse and Alcoholism heavy drinking defined as men >4 drinks on any day or 14 per week and women >3 drinks on any day or 7 per week, assessed using the Timeline Followback Method [38]

^§^Advanced Fibrosis defined as defined as a dichotomous outcome with positive fibrosis as a Fibrosis-4 (FIB-4) score >3.25, or FIB-4≥1.45 and ≤3.25 (consistent with possible fibrosis) with one of the following: 1) elastography (Fibroscan) suggestive of advanced liver fibrosis (≥10.5 kpa) or 2) AST to Platelet Ratio Index (APRI) ≥1.5

^§§^p<0.05

### Zinc deficiency and advanced fibrosis

The prevalence of advanced liver fibrosis was similar (27.7% vs. 23.0%, p = 0.47) among those with zinc deficiency compared to those with normal zinc levels. We did not detect an association between zinc deficiency and advanced liver fibrosis in either the partially adjusted model (adjusted odds ratio [aOR]: 1.25, 95% confidence interval [CI]: 0.62–2.53, p = 0.54) or the fully adjusted final regression model (aOR: 1.28, 95% CI: 0.62–2.61, p = 0.51) (Table 2). Furthermore, no significant association was found between zinc level categorized by tertiles and advanced liver fibrosis (lowest vs. highest zinc level tertile: aOR 1.32, 95% CI: 0.56–3.12, p = 0.52; middle vs. highest zinc level tertile: aOR 1.29, 95% CI: 0.55–3.04, p = 0.56) (Table 3). Among the covariates, only CD4 count <350 cells/μl was significantly associated with advanced liver fibrosis (aOR, 2.2, 95% CI: 1.05–4.62, p = 0.04) in the fully adjusted model. No significant association was found between continuous zinc level and FIB-4 score using either the partially (adjusted mean difference (AMD) in FIB-4 score per 1 unit increase in zinc level: -0.51, 95% CI: -3.20–2.18, p = 0.71) or fully adjusted linear regression models (AMD per 1 unit increase in zinc level: -0.55, 95% CI: -3.24–2.13, p = 0.29).

**Table 2:** Association between zinc deficiency and advanced liver fibrosis, logistic regression models.

	Partially adjusted model^†^		Fully adjusted model^‡^	
	Adjusted Odds ratio (95% CI)	*p* value	Adjusted Odds ratio (95% CI)	*p* value
Zinc deficiency^§^	1.25 (0.62–2.53)	0.54	1.28 (0.62–2.61)	0.51
Time (years) since HIV diagnosis^¶^	1.02 (0.95–1.10)	0.57	1.02 (0.95–1.10)	0.61
Alcohol abuse or dependence	1.08 (0.37–3.22)	0.89	1.19 (0.37–3.85)	0.77
HIV viral load (Log_10_ IU/mL)^¶^	1.15 (0.84–1.60)	0.39	1.14 (0.83–1.56)	0.41
CD4 count				
<350	2.31 (1.11–4.80)	0.02	2.20 (1.05–4.62)	0.04
350–500	0.46 (0.16–1.33)	0.15	0.44 (0.15–1.30)	0.14
>500 (Reference Group)	1	—	1.00	—
Age^¶^	—	—	0.98 (0.92–1.05)	0.61
Gender	—	—	0.70 (0.31–1.61)	0.40
Hepatitis B	—	—	1.42 (0.70–2.91)	0.33
BMI^¶^	—	—	0.99 (0.88–1.10)	0.83

BMI, body mass index

^†^Adjusted for time since HIV diagnosis, alcohol abuse or dependence, log_10_ HIV viral load, and CD4 count.

^‡^Adjusted for age, gender, hepatitis B co-infection, BMI, time since HIV diagnosis, alcohol abuse or dependence, log_10_ HIV viral load, and CD4 count.

^§^Zinc deficiency defined as concentration <0.75 mg/L.

^¶^Results reported per 1-unit increase.

**Table 3:** Association between zinc tertile level and advanced liver fibrosis, logistic regression models.

	Partially adjusted model^†^		Fully adjusted model^‡^	
	Adjusted odds ratio (95% CI)	*p* value	Adjusted odds ratio (95% CI)	*p* value
Zinc level^§^				
Lowest zinc level tertile	1.33 (0.57–3.11)	0.51	1.33 (0.56–3.12)	0.52
Middle zinc level tertile	1.37 (0.59–3.16)	0.47	1.29 (0.55–3.04)	0.56
Highest zinc level tertile (Reference)	1	—	1	—
Time (years) since HIV diagnosis^¶^	1.02 (0.95–1.10)	0.55	1.02 (0.95–1.10)	0.59
Alcohol abuse or dependence	1.07 (0.36–3.19)	0.90	1.17 (0.36–3.80)	0.79
HIV viral load (Log_10_ IU/mL)^¶^	1.14 (0.84–1.55)	0.41	1.13 (0.83–1.54)	0.44
CD4 count				
<350	2.27 (1.09–4.74)	0.03	2.19 (1.05–4.60)	0.04
350–500	0.46 (0.16–1.36)	0.16	0.45 (0.15–1.33)	0.15
>500 (Reference)	1	—	1	—
Age^¶^	—	—	0.982 (0.92–1.05)	0.60
Gender	—	—	0.72 (0.31–1.65)	0.43
Hepatitis B	—	—	1.38 (0.67–2.83)	0.38
BMI^¶^	—	—	0.99 (0.88–1.10)	0.80

BMI, body mass index

^†^Adjusted for time since HIV diagnosis, alcohol abuse or dependence, log_10_ HIV viral load, and CD4 count.

^‡^Adjusted for age, gender, hepatitis B co-infection, BMI, time since HIV diagnosis, alcohol abuse or dependence, log_10_ HIV viral load, and CD4 count.

^§^ Lowest tertile: zinc concentration ≤0.7633 mg/L; middle tertile: zinc concentration ≥0.7646 mg/L and ≤0.9740 mg/L; highest tertile: zinc concentration >0.9740 mg/L.

^¶^Results reported per 1-unit increase.

## Discussion

In this cross-sectional analysis of a cohort of ART-naïve HIV/HCV co-infected Russians with heavy drinking, we did not detect an association between zinc deficiency and advanced liver fibrosis. Zinc deficiency at baseline was noted in one-third of the participants and advanced liver fibrosis was noted in approximately one-quarter of participants. Fibrosis was observed in a slightly higher proportion of zinc deficient patients (28%) as compared to those with normal zinc levels (23%).

Previous in vitro and in vivo studies demonstrate that zinc affects the process of fibrosis. Zinc supplementation decreases the action of certain enzymes, specifically prolyl hydroxylase, which is essential for collagen formation and, thus the development of fibrosis [19]. In addition, higher mitochondrial oxidative stress has been observed in studies of zinc deficiency [23]. These two factors along with increases in inflammation in the setting of zinc deficiency are thought to explain, in part, the hypothesized association of zinc deficiency and advanced liver fibrosis.

The present study’s findings differ from prior work. The present study’s findings differ from prior work. For example, the mean zinc level in our study was 0.9mg/L ± 0.30 mg/L. This is higher compared to an established cohort of HCV/HIV co-infected persons with and without advanced liver disease (0.62 mg/L ± 0.17 mg/L) [23], but was similar to a Polish cohort with alcoholic cirrhosis (0.96 mg/L ± 0.24 mg/L to 1.04 mg/L ± 0.34mg/L) [30]. Our study differed from others as well with regard to demographic characteristics. For example, Omran et al found that lower zinc levels were associated with advanced liver fibrosis in an older cohort (mean age 52) of HCV mono-infected Egyptian patients [31]. The serum zinc level among those with >F2 fibrosis was 0.52 mg/L ± 0.31 mg/L versus 0.63 ± 0.30 mg/L among those with ≤F2 fibrosis. Authors in that study used transient elastography and dichotomized patients with either mild to moderate fibrosis (≤F2, ≤7.3 kPa) or advanced fibrosis (>F2, >7.3 kPa), whereas we defined advanced liver fibrosis as FIB-4>3.25 or APRI≥1.5 or elastography≥10.5kPa. The two studies also differed in key demographic characteristics. Aside from being conducted in two countries with ethnic differences, our cohort was younger (mean age 33 versus 52), majority male (76% versus 42%), and had a lower BMI (22 vs 30). All of these factors have the potential to produce discrepant results.

Additionally, Martinez et al found that lower plasma zinc concentrations were associated with increases in FIB-4 scores in a cohort of HIV mono-infected and HCV co-infected persons [23]. Their longitudinal study (n = 487) analyzed zinc levels and FIB-4 scores over a median follow up of 34 months in a predominantly older (mean age 47) African American male population. Most participants were on ART with an undetectable HIV viral load and did not have concomitant substance use disorders; no participants reported injection drug use. Their study highlights the potential impact of zinc deficiency on liver fibrosis in the absence of multiple other factors that contribute to the progression of fibrosis (i.e., untreated HIV infection, heavy alcohol use), which were prevalent in our population. Also, this Russian study did not include black participants. Previous research suggests that there may be a moderating effect of race on the progression of fibrosis [32, 33]. Taken together, our results along with the findings of Martinez et al suggest that the protective effect of zinc is more prominent among persons without multiple risk factors for liver fibrosis and potentially in African Americans.

Our study should be interpreted in the context of its limitations. First, this is a cross sectional study so our knowledge of the duration of zinc deficiency is limited. For example, it is plausible that our participants did not have longstanding zinc deficiency so the effects of chronic deficiency would not have been captured. Future studies should longitudinally assess the “dose effect” that duration of zinc deficiency may have on liver fibrosis. Additionally, even if we had found an association between advanced fibrosis and zinc deficiency, the cross-sectional design limits our ability to establish a causal direction. Second, this patient population has many risk factors for liver fibrosis—untreated HIV, HCV, HBV, alcohol use and injection drug use—which are all the most important factors in the development of fibrosis. These other factors may limit our ability to detect a significant impact from zinc deficiency in this exploratory analysis. Nevertheless, the results can be useful in the design of future studies that are larger in scale. Third, as this is a younger study population, our results may not be generalizable to older individuals. Since older populations likely have a longer duration of HCV infection leading to an increased likelihood of advanced liver fibrosis [34, 35], those with zinc deficiency may have even more pronounced fibrosis. Fourth, we used the FIB-4 scores coupled with elastography only when FIB-4 results were in the indeterminate range. FIB-4 may lower accuracy for detecting liver fibrosis in alcoholic liver disease compared to nonalcoholic liver disease [36]. Thus, future studies may consider using more accurate noninvasive tests of fibrosis in this population. Fifth, this is a secondary data analysis that was not designed to evaluate the relationship between zinc deficiency and advanced liver fibrosis. Post-hoc power calculations indicate that with 23% of those with normal zinc levels having advanced liver fibrosis, our study would have approximately 80% power to detect an odds ratio as small as 2.6. It, therefore, appears that this study was underpowered to detect the magnitudes of association that were observed. It, therefore, appears that this study was underpowered to detect the magnitudes of association that were observed. To this end, even the associations with HCV, HIV, and HBV—all independent determinants of fibrosis—do not reach statistical significance, therefore it is difficult to detect the association with any other exposure for which the magnitude of the effect may be smaller. Thus, we view our study as exploratory in nature. Finally, we relied on self-reported history of alcohol use, time since HIV diagnosis, and other important covariates (e.g. cocaine use), which may be under reported but may have independent effects on either zinc concentration or liver fibrosis. This is a problem that others have encountered during studies of liver fibrosis and alcohol-induced liver disease among HCV/HIV-coinfected persons [37]. These limitations notwithstanding, this study serves to expand the existing literature on the effects of zinc, an essential nutrient, in a unique population.

In conclusion, in this study of young ART-naïve HIV/HCV co-infected Russians most with heavy alcohol use, we did not detect an association between zinc deficiency and accelerated onset of advanced liver fibrosis. Future studies with larger sample sizes that examine the longitudinal association between chronic zinc deficiency and the rate of liver fibrosis progression among HCV-mono-infected and HIV/HCV co-infected persons would be of interest.

## Supporting information

S1 File A limited dataset is available in the supporting file “plosone_20190503.csv”. (CSV)
